# Oxytocin Attenuates Yohimbine-Induced Reinstatement of Alcohol-Seeking in Female Rats via the Central Amygdala

**DOI:** 10.3390/bs13070556

**Published:** 2023-07-04

**Authors:** Samantha M. Wilfur, Elizabeth C. McNeely, Aliya A. Lackan, Cassie P. Bowers, Kah-Chung Leong

**Affiliations:** Department of Psychology, Trinity University, San Antonio, TX 78212, USA; swilfur@trinity.edu (S.M.W.); emcneely@trinity.edu (E.C.M.); alackan@trinity.edu (A.A.L.); cbowers@trinity.edu (C.P.B.)

**Keywords:** oxytocin, females, yohimbine, alcohol, reinstatement, central amygdala, stress

## Abstract

Alcohol use disorder is a significant public health concern, further exacerbated by an increased risk of relapse due to stress. In addition, factors such as biological sex may contribute to the progression of addiction, as females are especially susceptible to stress-induced relapse. While there have been many studies surrounding potential pharmacological interventions for male stress-induced ethanol reinstatement, research regarding females is scarce. Recently, the neuropeptide oxytocin has gained interest as a possible pharmacological intervention for relapse. The present study examines how oxytocin affects yohimbine-induced reinstatement of ethanol-seeking in female rats using a self-administration paradigm. Adult female rats were trained to press a lever to access ethanol in daily self-administration sessions. Rats then underwent extinction training before a yohimbine-induced reinstatement test. Rats administered with yohimbine demonstrated significantly higher lever response indicating a reinstatement of ethanol-seeking behavior. Oxytocin administration, both systemically and directly into the central amygdala, attenuated the effect of yohimbine-induced reinstatement of ethanol-seeking behavior. The findings from this study establish that oxytocin is effective at attenuating alcohol-relapse behavior mediated by the pharmacological stressor yohimbine and that this effect is modulated by the central amygdala in females. This provides valuable insight regarding oxytocin’s potential therapeutic effect in female stress-induced alcohol relapse.

## 1. Introduction

According to the 2021 National Survey on Drug Use and Health, 29.5 million people in the United States suffered from alcohol use disorder (AUD) within the past year. This alarming statistic demonstrates the importance of understanding the factors contributing to the progression and relapse of AUD [1]. The majority of research thus far has focused on understanding this disorder in male subjects. However, recent evidence suggests that AUD prevalence is currently increasing at a much higher rate in women than in men [2] and that the gender gap in alcohol use is narrowing [3]. Among adolescents who started to drink between ages 11 and 14, women were found to progress to an episode of heavy drinking more quickly than men [4], and women are also generally more susceptible to alcohol-related injury, death, and other health issues [5]. Taken together, this evidence highlights the importance of further investigation of AUD using female subjects.

There are a number of factors that contribute to the development and relapse of AUD, including physiological factors, such as stress, which have consistently been found to influence this disorder [6]. Previous work has consistently demonstrated that stress and anxiety can increase alcohol consumption and the likelihood of relapse in abstinent individuals [7]. Females have been shown to generally be more susceptible to stress and anxiety than males due to biological differences in stress-related receptors, which is indicated by the higher prevalence of anxiety disorders in women than men [8,9]. Further, women report different motives behind their alcohol use than men; specifically, women have a higher likelihood of self-medicating their emotional distress, negative affect, mood disorders, and anxiety disorders by using alcohol [10]. Therefore, it is imperative to examine potential pharmacotherapeutic options to attenuate stress-induced alcohol-seeking behavior in females.

Recently, the neuropeptide oxytocin (OXT) has been shown to be an effective anxiolytic [11,12,13]. Several studies have determined that OXT reduces both the behavioral and physiological effects of anxiety in rodents [14]. Higher endogenous oxytocin levels were also shown to be correlated with lower anxiety levels in females [15]. In humans, anxiety levels were significantly reduced following acute intranasal administration of OXT [16]. In rodents, OXT diminished the behavioral consequences of social stress [17] and unpredictable stress [18]. Stress-related increases in corticosterone levels were subsequently reduced following OXT administration, and this corresponded with a decrease in stress-related behaviors [19]. Limited evidence has also found that OXT attenuated anxiety-induced reward-seeking behaviors [20,21]. 

The central amygdala (CeA) plays a vital role in emotional processes such as anxiety and stress and is also a critical structure in the emotional processing of drug-related stimuli by projecting to downstream effector regions [22]. Importantly, the CeA has also been found to be a key structure in mediating the effect of stress-induced drug-seeking behavior on several drugs of abuse, such as cocaine [23,24], nicotine [25], and ethanol [26]. OXT is primarily synthesized in the magnocellular neurons of the paraventricular nucleus and projects to the CeA, a region that has been found to express oxytocin receptors (OXTRs) [27,28]. Several studies have highlighted that the aforementioned anxiolytic effects of OXT may be mediated by OXT’s mechanism within the CeA, with the intra-CeA infusion of OXT demonstrating diminished fear responses [29] and endogenous release of OXT into the CeA showing suppression of fear expression [30]. Previously published work has demonstrated that OXT may attenuate stress-induced alcohol-seeking behavior in male rats [31], though the effect in females is still not known. 

The aim of the present study is to determine whether OXT attenuates stress-induced alcohol-seeking behavior in females and to identify whether the CeA is a key structure mediating this effect. We employed an ethanol self-administration and reinstatement paradigm in which we reinstated ethanol-seeking behavior using the anxiogenic drug yohimbine (YOH) [31]. We determined that systemically administered OXT effectively diminished the YOH-induced reinstatement of ethanol-seeking behavior in female rats. When OXT was infused directly into the CeA, we found that OXT attenuated YOH-induced reinstatement of ethanol-seeking behavior.

## 2. Materials and Methods

### 2.1. Animals

Adult female (maintained at 200–250 g throughout the study) Sprague-Dawley rats aged between 10 and 14 weeks (Charles River Laboratories, n = 18) were used in this study. Rats were single-housed on a reverse 12:12 h light-dark cycle in a set temperature and humidity-controlled vivarium. During the experiment, the animals were food restricted to 20 g of food daily in order to maintain a weight compatible with surgical procedures. Animals were water-restricted for 4 h prior to each conditioning session until operant conditioning was stabilized at >10 active lever presses per session. Rats received water ad libitum throughout the remainder of the day after each session. All procedures were approved by the Institutional Animal Care and Use Committee (IACUC; 082621-KCL) of Trinity University.

### 2.2. Apparatus

All self-administration, extinction, and reinstatement procedures were carried out in a metallic operant chamber (30.48 cm × 25.40 cm × 30.48 cm) containing a metal grid floor and an overhead activity counter (Coulbourn Instruments, Holliston, MA, USA). Each chamber contained a house light, two retractable levers (one active, one inactive) with cue lights above each lever, and an access-controlled optical lickometer behind a guillotine door. The lickometer was connected to a standard drinking tube and contained photocell sensors capable of measuring the number of licks each animal produced on the drinking tubes. The chamber was located inside a sound-attenuating cabinet with an ambient fan. All efforts were made to minimize any external smells or sounds, and the subjects only ran during the dark phase of their 12 h reverse light–dark cycle.

### 2.3. Drugs

Oxytocin (Cell Sciences, Newburyport, MA, USA) was dissolved in 0.9% NaCl saline and administered either intraperitoneally (i.p.; 1 mg/kg at a volume of 1 mL/kg) or infused intra-CeA (0.5 µg at a volume of 0.5 µL). Previously published studies, which show localized pharmacological effects within the rat CeA, were the basis of the volume of oxytocin selected for this study [23,32]. Yohimbine (Acros Organics, Carlsbad, CA, USA) was dissolved in water and administered intraperitoneally (i.p.; 2 mg/kg) at a volume of 0.5 mL/kg, which is a dose that has been shown to induce reinstatement behavior in previous studies [2]. The self-administered ethanol was diluted in water to a concentration of 20% *v*/*v*.

### 2.4. Surgery

Prior to behavioral training, the rats received implantations of guide cannulas extending into the CeA. The rats were injected with the antibiotic Cefazolin (0.2 mL; i.p.) and the analgesic Rimadyl (0.03 mL; i.p.; Zoetis, Kalamazoo, MI, USA) prior to intracranial surgery. The rats were then anesthetized with Isoflurane and mounted on a stereotaxic platform. Cannulae were bilaterally targeted above the CeA (−2.4 AP; ±4.0 ML; −6.9 DV from bregma). Once inserted, cannulae were secured in place with jeweler screws and dental acrylic cement. The same doses of Cefazolin and Rimadyl were injected intraperitoneally as post-operative care for 3 days following surgery. All surgical methods were performed using aseptic techniques, and the rats were given 5–7 days of recovery before any experimentation was performed.

### 2.5. Procedure

#### 2.5.1. Ethanol Habituation

All animals underwent a two-week ethanol habituation period prior to operant conditioning. Animals would receive ethanol (10% *v*/*v*) for 10 h via standard drinking bottles in their home cages, with no water provided. Immediately after ethanol exposure, animals would receive water ad libitum for 1 h, after which they were water deprived for 12 h. This was repeated for three consecutive days of each of the two habituation weeks, with the animals receiving water ad libitum for the remaining four days of the week. The animal and bottle weights were recorded before and after ethanol exposure to determine the amount of ethanol consumed. Ethanol consumption during the ethanol habituation period is presented in Table 1.

#### 2.5.2. Ethanol Self-Administration

Operant conditioning was conducted in daily 1 h sessions, during which a press on the active lever resulted in the opening of the lickometer guillotine door and access to the ethanol drinking tube. The access period lasted for 30 s or until the animal began ethanol (20% *v/v*) consumption, whichever occurred first. Upon initial consumption, animals were given an additional 5 s before the door closed. Pressing the active lever produced no consequence. Each animal started in FR1 (1 active lever press for one period of ethanol access). The criteria for completion of FR1 was set at 15 daily active lever presses for two consecutive days, after which animals moved to FR3 (3 active lever presses for one period of ethanol access). The criteria for completion of self-administration was set at 35 daily active lever presses for two consecutive days. Animals that did not meet the criteria after 21 sessions (n = 7) were removed from the study.

Following self-administration, the lever press response was extinguished in daily 1 h sessions, during which neither the presses on the active or inactive levers provided consequential access to the ethanol drinking tube. A minimum of 7 daily sessions and less than 20 presses on two consecutive days was required before stress-induced reinstatement testing, consistent with criteria in previous studies employing similar behavioral paradigms [33,34].

**Figure 1:**
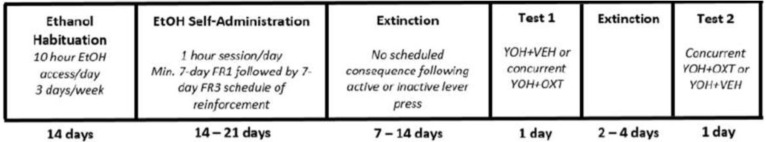
Timeline depicting the experimental paradigm across Experiments 1 and 2. All animals underwent ethanol habituation followed by ethanol self-administration, extinction, and reinstatement testing. YOH = yohimbine; VEH = vehicle; OXT = oxytocin.

The anxiogenic drug yohimbine (YOH; 2 mg/kg; i.p.) was administered 30 min prior to entering the chamber for stress-induced reinstatement testing. During testing, pressing the active and inactive levers did not provide access to ethanol. Each test session lasted 1 h, and each rat was tested twice, with at least two extinction sessions occurring between tests or until previous extinction criteria were met. Test conditions were counterbalanced across all animals, and the absence of a test order effect is confirmed by previous studies [35,36,37]. The experimental paradigm and timeline are represented in Figure 1.

#### 2.5.3. Experiment 1

To determine whether OXT administration effectively attenuated the ethanol-seeking behavior reinstated by the YOH, all rats underwent the self-administration, extinction, and reinstatement procedures described above. The rats in the Experiment 1 group received two reinstatement tests, which were randomly counterbalanced across all rats in this group and separated by at least two extinction trials between tests. In one reinstatement test, rats received concurrent injections of OXT (1 mg/kg; i.p.) and YOH (2 mg/kg; i.p.) (n = 9), and in the other, they received vehicle (VEH) and YOH (n = 9). As all animals received two injections during the test day, this accounted for the number of injections influencing reinstatement behavior. All drug administration occurred 30 min prior to the reinstatement testing to account for any locomotor effect of OXT [20,33,36].

#### 2.5.4. Experiment 2

To determine whether the CeA plays a role in mediating the attenuating effect of OXT on the ethanol-seeking behavior reinstated by the YOH, the rats underwent the self-administration, extinction, and reinstatement protocols described above. The rats in the Experiment 2 group also received two reinstatement tests, which were randomly counterbalanced across all rats in this group and separated by at least two extinction trials between tests. Here, the reinstatement tests consisted of either concurrent intra-CeA infusions of OXT (0.5 µg) and YOH injections (2 mg/kg; i.p.) (n = 9) or concurrent intra-CeA VEH infusions and YOH injections (2 mg/kg; i.p.) (n = 9). As with Experiment 1, all drug administration occurred 30 min prior to the reinstatement testing.

### 2.6. Tissue Collection and Histological Analysis

**Figure 2:**
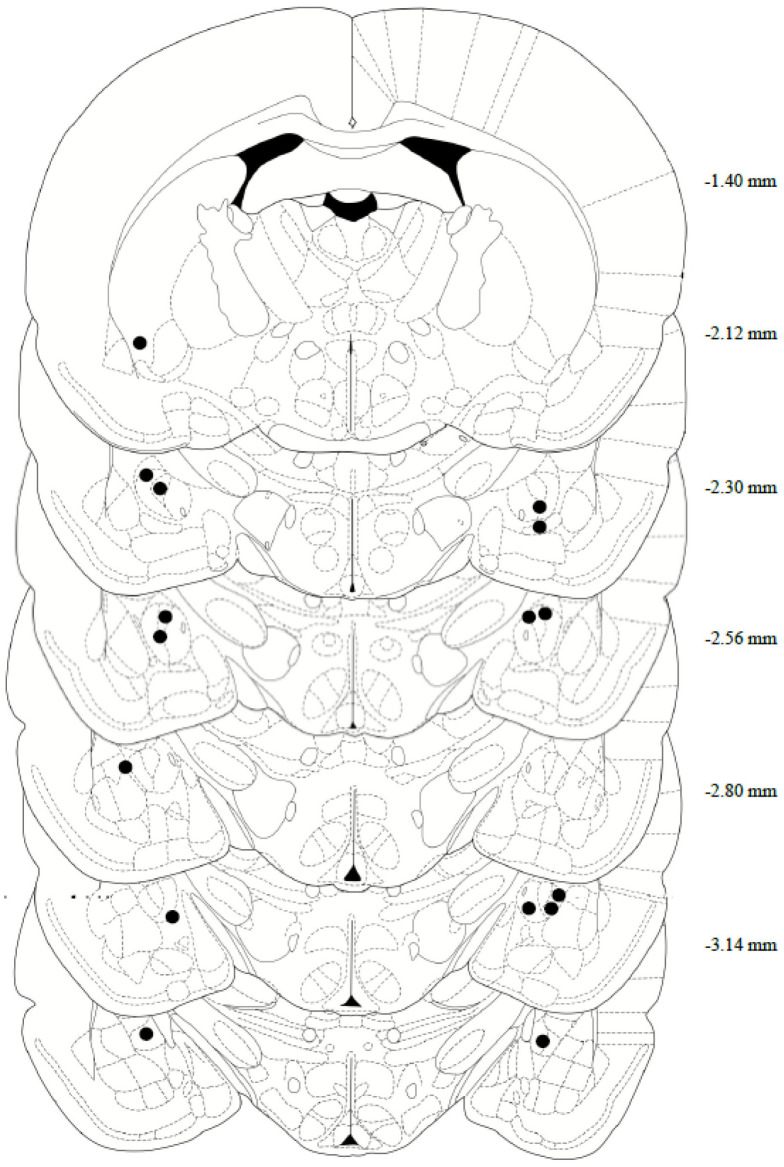
Anatomical depiction of terminal point of the internal cannulae injectors used for intra-central amygdala infusions. Numerical measurements indicate posterior coordinates relative to bregma. All cannulae were targeted toward the CeA (−2.4 AP; ±4.0 ML; −6.9 DV from bregma).

After completing both reinstatement tests, the rats’ brains were collected for histological assessment of the cannula placement. In brief, the rats were deeply anesthetized with phenytoin/pentobarbital, then transcardially perfused with 150–200 mL of cold 0.9% PBS, followed by 200–300 mL of 10% formalin. The rats were decapitated, then the brains were removed and post-fixed in 10% formalin for 24 h, submerged in 20% sucrose for 48 h, and then sectioned on a cryostat at 40 μm and collected on microscope slides. Tissue slices were stained with Cresyl Violet, and a CeA internal cannula placement was confirmed using the rat brain in Stereotaxic Coordinates Atlas, 7th edition (Figure 2). Two animals were removed from the study following confirmation of missed cannula placements.

### 2.7. Data Analysis

A two-way repeated measures ANOVA was performed to determine the acquisition of operant lever-pressing behavior during self-administration. A repeated measures one-way ANOVA was used to evaluate the effects of OXT administration on the YOH-induced reinstatement of ethanol-seeking behavior by analyzing the differences in lever presses between OXT + YOH and VEH + YOH groups, compared to their lever-pressing behavior over the last two days of extinction (EXT). Pairwise comparisons were conducted using Dunnett’s multiple comparisons tests. All data were presented as the mean ± S.E.M., and α was set at *p* < 0.05.

## 3. Results

### 3.1. Systemic Administration of Oxytocin Attenuates Yohimbine-Induced Alcohol-Seeking in Female Rats

**Figure 3:**
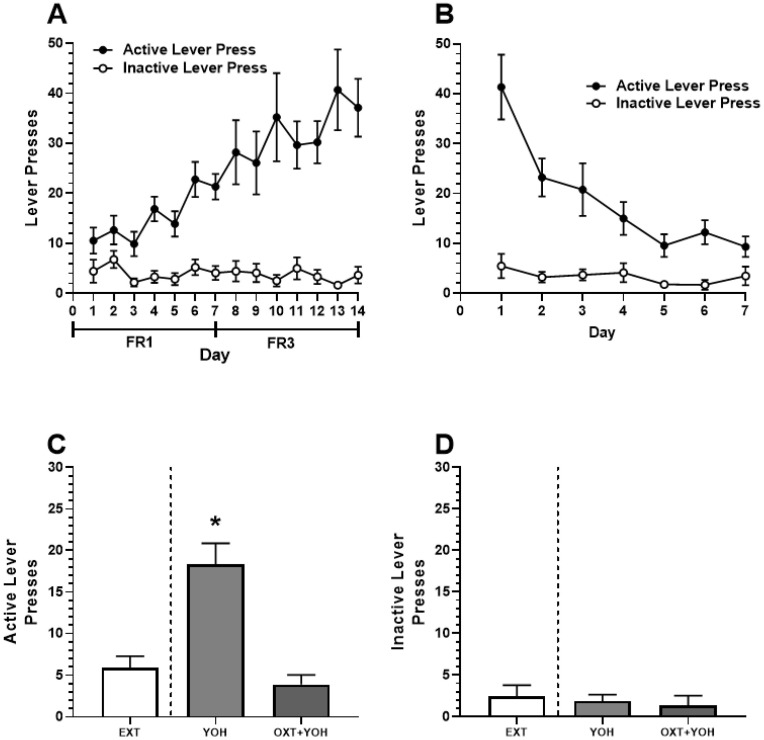
(**A**) Experiment 1: Active and inactive presses during ethanol self-administration across FR1 and FR3 schedules of reinforcement. (**B**) Active and inactive presses during extinction. (**C**) Systemic administration of OXT-attenuated YOH-induced reinstatement of ethanol-seeking behavior. YOH administration alone results in the reinstatement of lever-pressing behavior. Concurrent administration of YOH and OXT results in diminished active lever pressing. (**D**) YOH alone or concurrent YOH and OXT did not have any effect on inactive lever presses. * denotes a significant difference in active lever presses relative to EXT (avg. lever presses across the last 2 days of extinction) at *p* < 0.05. YOH = yohimbine; OXT = oxytocin.

In Experiment 1, all female rats demonstrated successful acquisition of alcohol self-administration. A two-way ANOVA revealed a significant main effect of Day [F(13,208) = 5.00, *p* < 0.01] and Active Lever [F(1,16) = 41.87, *p* < 0.01]. A significant Day x Lever interaction was also revealed [F(13, 208) = 5.87, *p* < 0.01]. A post hoc Dunnett’s multiple comparisons test revealed that rats demonstrated significantly more active lever pressing for each of the last 7 days of self-administration relative to Day 1 (*p* < 0.01). No increase in inactive lever pressing was found (n.s.; Figure 3A). Successful extinction was demonstrated following self-administration. A two-way ANOVA comparing lever pressing during extinction revealed a significant main effect of Day [F(6,96) = 13.33, *p* < 0.01], Lever [F(1,16) = 28.04, *p* < 0.01], and a significant Day x Lever interaction [F(6,96) = 9.29, *p* < 0.01]. A post hoc Dunnett’s multiple comparisons test revealed a significant reduction in active lever pressing for every subsequent day relative to Day 1 (*p* < 0.05). No significant differences in inactive lever pressing were observed (n.s.; Figure 3B).

On test day, repeated measures of one-way ANOVA revealed a significant difference in the treatment group [F(1.60, 12.74) = 39.39, *p* < 0.001] between active lever pressing during the last two days of extinction, YOH+OXT, and YOH+VEH groups. A post hoc Dunnett’s multiple comparisons test revealed that animals receiving YOH+VEH during test day demonstrated significantly higher active lever presses (M = 18.33) relative to EXT (M = 5.89; *p* < 0.01). Animals receiving YOH+OXT did not display any significant differences in active lever pressing (M = 3.78) relative to EXT (M = 5.89, n.s.). A repeated measures one-way ANOVA did not reveal any significant difference between treatment groups for inactive lever presses during the test [F(1.39, 11.18) = 1.77, n.s.]. These results suggest that administration of yohimbine significantly increased active lever pressing during test day while concurrent administration of oxytocin with yohimbine attenuated this effect (Figure 3C).

### 3.2. Intra-CeA Infusion of Oxytocin Attenuates Yohimbine-Induced Alcohol-Seeking in Female Rats

**Figure 4:**
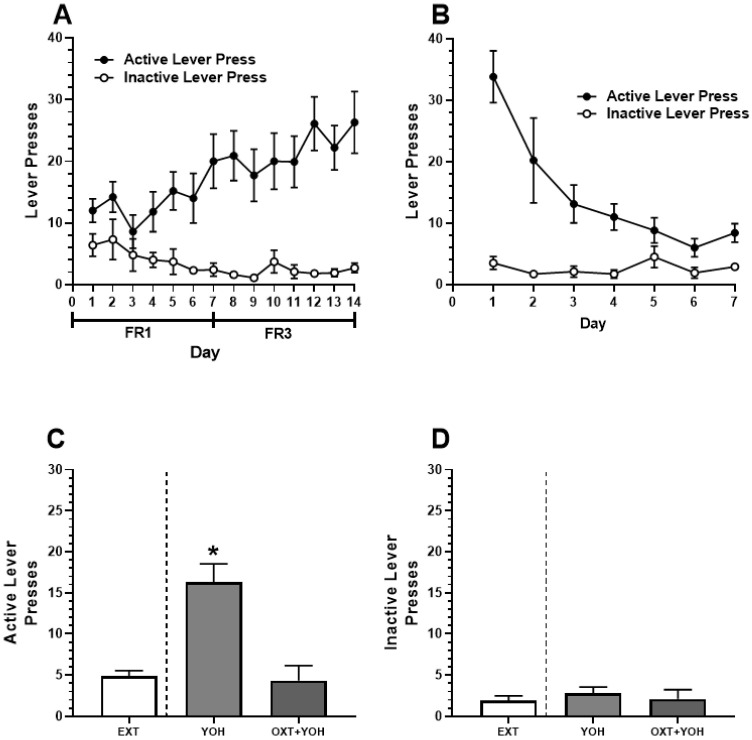
(**A**) Experiment 2: Active and inactive presses during ethanol self-administration across FR1 and FR3 schedules of reinforcement. (**B**) Active and inactive presses during extinction. (**C**) Intra-CeA administration of OXT-attenuated YOH-induced reinstatement of ethanol-seeking behavior. YOH administration alone results in the reinstatement of lever-pressing behavior. Consecutive administration of YOH and OXT results in diminished active lever pressing. (**D**) YOH alone or concurrent YOH and OXT did not have any effect on inactive lever presses. * denotes a significant difference in active lever presses relative to EXT (avg. lever presses across the last 2 days of extinction) at *p* < 0.05.

All animals in Experiment 2 demonstrated successful acquisition of alcohol self-administration. A two-way ANOVA revealed a significant Day x Lever interaction [F(13, 234) = 2.85, *p* < 0.01]. A post hoc Dunnett’s multiple comparisons test comparing lever presses on each day of self-administration to Day 1 revealed that rats demonstrated significantly more active lever pressing on the last day of self-administration (M = 26.30) relative to Day 1 (M = 12.00; *p* < 0.01). No increase in inactive lever pressing was found when comparing the last day of self-administration (M = 2.7) to Day 1 (M = 6.4, n.s.; Figure 4A). Successful extinction was demonstrated following self-administration. A two-way ANOVA revealed a significant Day x Lever interaction [F(6,108) = 7.35, *p* < 0.01]. A post hoc Dunnett’s multiple comparisons test revealed a significant reduction in active lever pressing when comparing the last day of extinction (M = 8.4) to extinction day 1 (M = 33.80; *p* < 0.01). No significant differences in inactive lever pressing were observed between the first (M = 3.50) and the last day of extinction (M = 2.90; n.s.; Figure 4B).

On the test day, repeated measures of one-way ANOVA comparing active lever presses revealed a significant difference in the treatment group [F(1.76, 14.11) = 21.63, *p* < 0.01] between presses during the last two days of extinction, YOH + intra-CEA OXT, and YOH + intra-CeA VEH groups. A post hoc Dunnett’s multiple comparisons test revealed that animals receiving YOH + intra-CeA VEH during test day demonstrated significantly higher active lever presses (M = 16.33) relative to EXT (M = 4.89; *p* < 0.01). Animals receiving YOH+OXT did not display any significant differences in active lever pressing (M = 4.33) relative to EXT (M = 4.89, n.s.). A repeated measures one-way ANOVA did not reveal any significant difference between treatment groups for inactive lever presses during the test [F(1.49, 11.50) = 0.39, n.s.]. These results suggest that direct infusion of OXT into the CeA diminished yohimbine-induced alcohol-seeking behavior in female rats, highlighting OXT’s potential mechanism within this structure (Figure 4C).

## 4. Discussion

The present study suggests that oxytocin, administered either peripherally or centrally into the CeA, attenuates yohimbine-induced ethanol-seeking behavior in female rats. When administered oxytocin, female rats demonstrated diminished yohimbine-induced active lever pressing during reinstatement testing, suggesting a decreased effect of yohimbine on alcohol-seeking behavior. The present study adds to a growing body of literature demonstrating that OXT is an effective modulator of alcohol-reward processes and behaviors. Systemic OXT administration has been found to diminish alcohol withdrawal symptoms [38] as well as various behavioral effects of alcohol exposure [39,40]. OXT also decreases preference and intake of alcohol in various rodent models [41,42]. OXT has been shown to reduce alcohol self-administration and consumption [43]. In addition, OXT reduces cue reactivity to alcohol-associated cues in rats and humans [44]. Our findings may extend to the use of oxytocin as a potential therapeutic target for the intervention of stress-induced ethanol-seeking behavior in females, which is consistent with the literature which has previously shown that OXT is effective in attenuating stress-induced alcohol-seeking behavior in male rats [31] as well as male and female mice [45]. Importantly, consistent with previous studies, this study highlights that the CeA is a key structure mediating this effect of oxytocin, as previously demonstrated in male rats [29]. Our results offer insight into the use of OXT as an effective anxiolytic to diminish the effect of stress on alcohol addiction-related behaviors. Previous studies have shown that OXT may have anxiolytic effects in animals [30,46] and humans [47,48]. For example, intranasal administration of OXT reduces anxiety related to public speaking situations in humans [11], and chronic intranasal administration may alleviate exaggerated threat reactivity in subjects with elevated anxiety levels [49]. Peripheral administration of OXT reduces anxiety-like behaviors of animals when placed in novel environments [46] and diminishes the overall anxiety-related behaviors across several behavioral tests of anxiety [50]. Furthermore, stimulation of endogenous oxytocin release significantly attenuated freezing behavior in a fear conditioning paradigm [30]. Overall, the results from the present study further emphasize the use of OXT as an anxiolytic peptide and expand its applications into diminishing the effects of stress-induced reinstatement of alcohol-seeking behavior. However, as noted above, the administration of OXT has been found to attenuate alcohol self-administration and intake [41,42] in the absence of stress effects. It is certainly possible that the effect of OXT demonstrated in this study may not be limited to attenuating yohimbine’s effect on alcohol-seeking reinstatement and instead may be attenuating alcohol-seeking in other capacities, such as motivation or alcohol-related cue reactivity. This should certainly be taken into consideration within the context of the present findings. However, the specific yohimbine-induced paradigm utilized in this study increases the likelihood that the results presented here offer an opportunity to view OXT as a potential therapeutic target for stress-induced reinstatement of drug-seeking behavior.

The findings discussed here are consistent with previous studies showing that OXT successfully attenuates stress-induced ethanol-seeking behavior [31,45]. Similar to results found in males [31], the present study also identifies the CeA as a key structure important for mediating OXT’s attenuating effect on stress-induced reinstatement of alcohol-seeking in female rats. These findings add to the current body of literature suggesting that OXT produces anxiolytic effects via mechanisms within the CeA [29,30]. Though the present study did not identify the specific mechanism through which OXT may be attenuating stress-induced alcohol-seeking behavior via the CeA, previous studies have demonstrated a high density of oxytocin receptors (OXTRs) found within the CeA on GABAergic interneurons [27,51,52] and astrocytes [53], both of which modulate GABAergic output of the CeA. Given that the CeA in both males and females has been shown to be physiologically similar with a similar expression of OXTRs and downstream projections [54], it is likely that the consistent results found in the present study in female rats and previously in male rats [31] may suggest a similar underlying mechanism of OXT on stress-induced ethanol-seeking behavior. The volume of OXT infusion (0.5 µL) was selected based on published work that confirmed an isolated effect of OXT in CeA on emotional [55] and social learning [54]. However, it is still possible that OXT infusion may not be localized to the CeA. Previously published work by Huber et al. (2005) has demonstrated that OXTRs are primarily expressed in rat CeA rather than adjacent amygdalar nuclei [56]. Similarly, neurophysiological changes in neuronal excitability mediated by OXTRs appear localized to CeA neuronal populations [28]. Despite this, the possible diffusion and spread of OXT should continue to be taken into consideration.

It is possible that sex differences may play a role in alcohol-seeking behavior and the effect that OXT produces on this behavior. Previous studies have found that the OXT system may interact differently with alcohol usage in males versus females. A decrease in OXT and an increase in OXTR binding sites were found in male alcohol-dependent rats and male alcohol-dependent patients compared to healthy controls, but no change was found in female subjects [57]; additionally, intranasal and intraperitoneal OXT administration both selectively inhibited alcohol-seeking in male, but not female, prairie voles [58]. In humans, the OXTR rs53576 polymorphism, which is suggested to decrease oxytocinergic functioning, was associated with increased alcohol use in males but not females [59]. These studies suggest that the OXT system and alcohol may be more strongly linked in males. However, other studies have also found that OXT administration effectively attenuates stress-induced reinstatement in both male and female mice [45] and that in OXTR knockout mice, alcohol consumption was only increased in female, but not male, subjects [60], which reveals inconsistencies in the literature regarding differential interactions between alcohol usage and OXT in males versus females. The results of the present study, which demonstrate that yohimbine-induced alcohol-seeking behavior in females may be attenuated by intraperitoneal or intra-CeA OXT administration, are therefore extremely valuable when compared with the previous literature; taken together, the findings of these studies highlight the importance of continuing to investigate the role of OXT in alcohol-seeking in females, especially in comparison to male subjects.

The present study utilizes the alpha-2 adrenoceptor antagonist, yohimbine, as a pharmacological stressor consistent with its use in previous studies to effectively induce physiological stress [61,62]. Various studies have since employed YOH as a pharmacological stressor to drive stress-induced reinstatement to various drugs of abuse [63,64,65]. YOH provides an appropriate method of inducing stress as it offers less variability than other types of stressors, such as foot shock [63,65]. However, it should be noted that YOH’s physiological effects are not limited to stress. YOH has been found to increase perseverative responding [66] and motor behaviors [67]. There is also limited evidence suggesting that YOH’s effects may not be limited to norepinephrine release and also increases serotonergic release [68]. Our data suggest that YOH did not simply increase lever pressing through increased locomotor behavior, as active and inactive lever pressing during reinstatement tests demonstrated increased active lever presses only (Figure 3C,D and Figure 4C,D). While we did not directly test whether YOH drives stress response in this study, human studies have provided support that YOH induces physiological stress [69,70]. Regardless, it is important to consider that while YOH induces alcohol reinstatement behavior in females, as shown here, it may not necessarily be through stress-related processes.

Overall, the present study demonstrates that OXT is an effective neuropeptide in attenuating stress-induced alcohol-seeking behavior in female rats. Furthermore, the CeA continues to be a key structure mediating this attenuating effect on OXT. This study adds to a growing body of research investigating OXT as a potential pharmacotherapeutic intervention for various drug-seeking behaviors.

## Figures and Tables

**Table 1:** Means and Standard Deviations for Ethanol Consumption during Ethanol Habituation.

Ethanol Consumption (g/kg)	Experiment 1		Experiment 2	
	*M*	*SD*	*M*	*SD*
Day 1	6.46	1.40	8.55	2.34
Day 2	6.59	0.89	8.16	3.02
Day 3	7.52	1.29	8.94	2.88
Day 4	4.89	2.14	8.60	3.65
Day 5	7.74	2.39	9.12	4.41
Day 6	9.31	2.32	8.99	3.11

Note. EtoH intake recorded over 10 h sessions each day. All animals received 3 consecutive days of EtOH habituation per week over two weeks.

## Data Availability

The data presented in this study are available in the present article and on request from the corresponding author.
